# A review of the *Delia interflua* group with descriptions of two new species (Diptera, Anthomyiidae)

**DOI:** 10.3897/zookeys.764.22736

**Published:** 2018-06-05

**Authors:** Jing Du, Wanqi Xue

**Affiliations:** 1 College of Life Science,; 2 Shenyang Normal University, Shenyang 110034, China; 3 Institute of Entomology, Shenyang Normal University, Shenyang 110034, China

**Keywords:** Anthomyiidae, *Delia*, key, new species

## Abstract

The *Delia interflua* group is reviewed to include 16 species. Two new species are described, namely *Delia subnemostylata* Xue & Du, **sp. n.**, *Delia nigeriposticrus* Xue & Du, **sp. n.** A catalogue and amended key to new species of this group are also included.

## Introduction

The genus *Delia* Robineau-Desvoidy, 1830 belongs to the dipteran family Anthomyiidae. More than 310 species in this genus (excluding Neotropical species) are known from around the world (Hennig1974, Ackland and Pont 1977, Fan et al. 1988, Pont 1989, Griffiths 1991, Dely-Draskovits 1993, Wei et al. 1996, Ackland 2008, Xue and Du 2008, Xue and Du 2009, Wang et al. 2014, Du and Xue 2017). The *Delia interflua* group is a small group and can be recognized by the following characters: the apices of the sternite V processes dilate and rounded, except for *D. subnemostylata* sp. n.; cercal plate with long setae; surstyli elongated, except for *D. kullensis*; usually the acrophallus is supported by a sclerotized bridge between the bases of the free paraphallic processes; the sclerotized bridge is prolonged downwards, forming a membranous process (Griffiths 1991, Xue and Du 2008). In this work, two new species of this group are described, *Delia subnemostylata* Xue & Du, sp. n., *Delia nigeriposticrus* Xue & Du, sp. n. The key produced by Wei et al. (1996) is here modified to include three couplets leading to the identification of the new species.

## Materials and methods

All specimens were collected from Yunnan province of China. Type specimens are deposited in the Diptera collection of the Institute of Entomology, Shenyang Normal University (**IESNU**). They were examined under an Olympus SZ-ST stereomicroscope. Morphological terminology is based mainly on that of McAlpine (1981). Abbreviations for terms used in this study are:

*a* anterior setae;

*acr* acrostichal setae;

*ad* anterodorsal setae;

*av* anteroventral setae;

*d* dorsal setae;

*dc* dorsocentral setae;

*ial* intra-alar setae;

*p* posterior setae;

*pd* posterodorsal setae;

*post acr* postsutural acrostichal setae;

*post dc* postsuturaldorsocentral setae;

*pra* prealar setae;

*prst acr* presutural acrostichal setae;

*prst dc* presutural dorsocentral setae;

*pv* posteroventral setae; and

R_4+5_ branch of radius.

### Addendum to the key by Wei et al. (1996) for the new species of the *Delia interflua* group (males)

**Table:** 

7a	Hind tibia with 2 rows of *pv*	***D. duplicipectina* Fan in Fan & Zheng, 1993**
–	Hind tibia with no more than 1 row of *pv*	**7b**
7b	Mid tibia without *pv*	***D. subnemostylata* Xue & Du, sp. n.**
–	Mid tibia with 2 *pv*	**7c**
7c	Hind tibia with 2–3*av*, 5*ad*, 5–7*pd* and 5*pv*	***D. nemostylata* Deng & Li, 1984**
–	Hind tibia with a row of *av* (approx. 9–10), a row of *ad* (approx. 7–8), a row of *pd* (3 strong) and a complete row of *pv*	***Delia nigeriposticrus* Xue & Du, sp. n.**

## Taxonomy

### Delia subnemostylata

Taxon classificationAnimaliaDipteraAnthomyiidae

Xue & Du sp. n.

http://zoobank.org/7B62520A-3712-4E81-83CE-A71C0897DFED

Figure 1

#### Type material.

***Holotype.*** China, Yunnan Province, Baimang Snowberg, 3800–4200 m, 5 July 2006, Mingfu Wang Co., ♂ (IESNU). ***Paratype***. China, same data as holotype, 3 ♂♂.

#### Diagnosis.

Frontal setae 4–5 pairs, *pra* approx. 1.2 times as long as posterior notopleural seta; sternite I with dense long fringes, sternite III with dense long setae; postgonite without setae.

#### Description.

***Holotype male.*** Body length 5.5 mm.

*Head*. Eyes only with several short ciliae in lower margin; frontal vitta red brown in lower part, remaining black, 2.0 times as wide as fronto-orbital plate; frons 1.5–2.0 times as wide as anterior ocellus; frontal vitta with a pair of inter frontal setae; without orbital setae; frontal setae 4–5 pairs, situated on lower half; fronto-orbital plate, parafacial with dark gray tomentum, parafacial 1.3 times as wide as postpedicel; antenna black, postpedicel 2.0–2.3 times as long as broad; arista pubescent, the longest aristal hairs shorter than its basal diameter; lower facial margin not projecting, vibrissal angle and frontal angle in the same vertical plane in profile; gena sparsely with dark gray tomentum, genal height approx. 1/4 of eye height; anterior margin of gena with two rows of upcurved subvibrissal setulae; postocular setae extending to ventral surface, epicephalon haired; proboscis short, prementum with gray tomentum sparsely, 2.5 times as long as broad, palpus black, equal to the length of prementum.

*Thorax*. Ground color black with fuscous tomentum; scutum with three distinct black vittae; with two rows of hair-like *prstacr*, only one pair of *post acr* distinctly in front of scutoscutellar suture, *dc* 2+3, *ial* 0+2; without outer posthumeral seta; *pra* approx. 1.2 times as long as posterior notopleural seta; scutellum without spots, lower surface with some pale hairs apically; anterior anepisternal setae absent; notopleuron haired; basisternum of prosternum, anepimeron, meron and katepimeron all bare; both anterior and posterior spiracles small and fuscous; katepisternal seta 1+2.

*Wing*. Base brown and basicosta fuscous; costa setulose only basally on ventral surface, anterior surface with a row of pectinated spines; costal spine subequal to crossvein r-m; radial node bare, calypters yellowish, lower calypter approx. 2/5 length of upper one; halter yellow.

*Legs*. Entirely black; fore tibia with 1 submedial *ad* and 1 medial *p*; mid femur without distinct *av*, with 4–5 strong *pv* in basal half; mid tibia with 1 submedial *av*, 1 *pd* and 1 preapical *p*, without *pv*; mid tarsomere 1 without distinct setae; hind femur with 7–8 *av* in distal 2/3 (2 strong), only with distinct *pv* in distal part; hind tibia with 3 *av*, 2 *ad*, 3 *pd*, and 4–5 *pv*; all tarsi shorter than tibiae, claws and pulvilli shorter than tarsomere 5.

**Figure 1.**
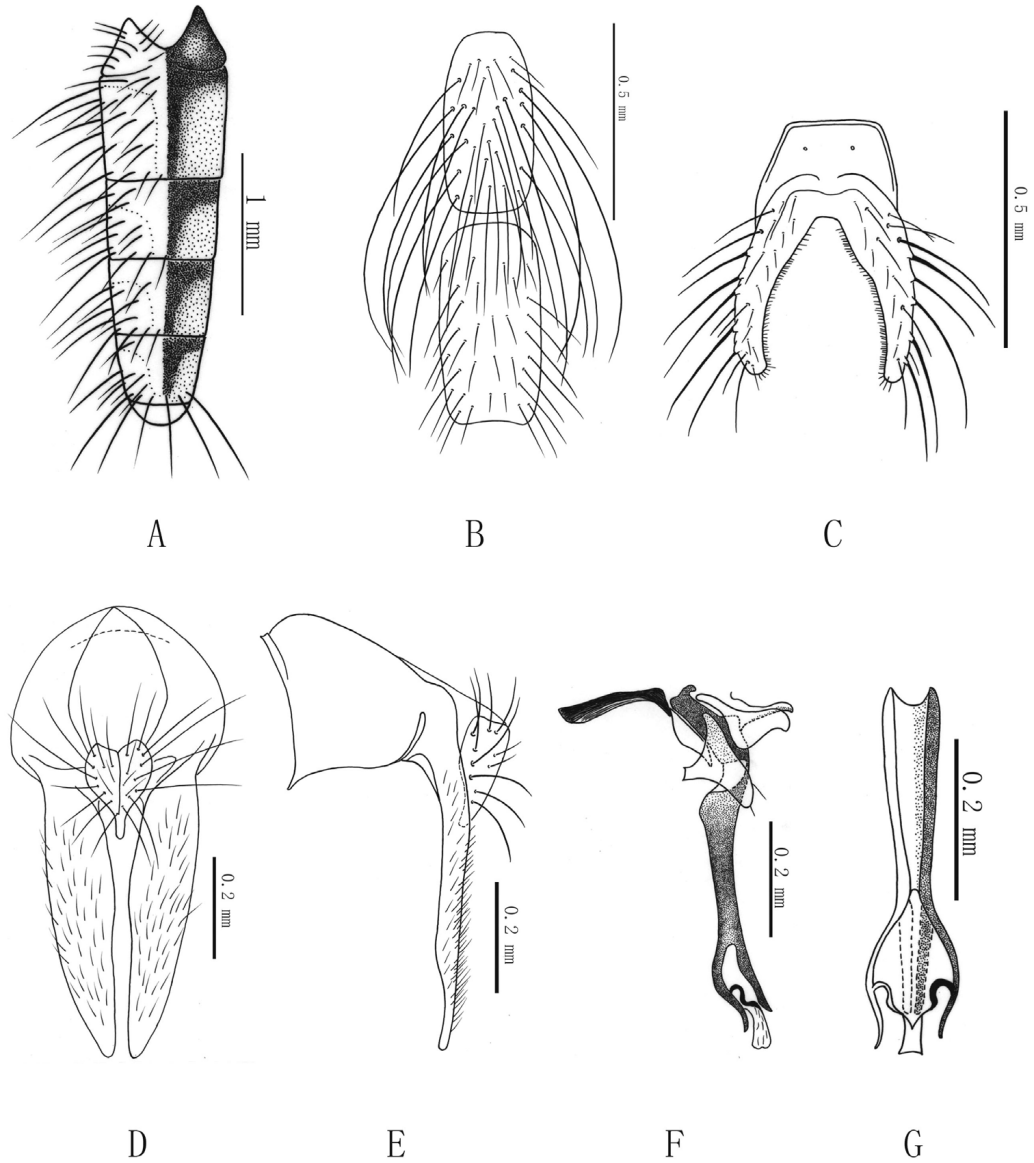
*Delia subnemostylata* Xue & Du, sp. n. (male). **A** Abdomen in dorsal view **B** Sternites III and IV in ventral view **C** Sternite V in ventral view **D** Epandrium, cerci, and surstyli in posterior view **E** Ditto, left lateral view **F** The hypandrial complex in left lateral view **G**. Distiphallus in anterior view.

*Abdomen*. Black, long flat-shaped(Fig. 1A); tergite 2 slightly longer; all tergites with T-shaped spots, lateral setae and posterior marginal setae developed, tergite VI bare; sternite I with dense and long fringes, sternite III with dense and long setae (Fig. 1B); sternite IV with short setae; sternite V processes (Fig. 1C) longer than base; cercal plate (Fig. 1D) 1.2 times longer than wide, heart-shaped, apex finger-shaped, bearing a few short setae; surstyli 3.0 times length of cercal plate, in lateral view (Fig. 1E) slender; pregonite with two setae, postgonite without setae. Aedeagus as illustrated in Figure 1F, G; acrophallus longitudinally directed, supported by a sclerotized bridge between the bases of the free paraphallic processes, the sclerotized bridge is prolonged downwards, forming a membranous process.

***Female.*** Unknown.

#### Remarks.

This new species is similar to *D. nemostylata* Deng & Li, 1984 as it has very similar genitalia, but differs from it for its frontal setae 4–5 pairs, katepisternal seta 1+2; lower calypter approx. 2/5 length of upper one; fore tibia with one submedial *ad* and one medial *p*; mid tibia without *pv*; hind tibia with two *ad* and three *pd*.

#### Etymology.

This new species is similar to *D. nemostylata* Deng & Li, 1984. Hence, its epithet is derived to reflect this relationship.

#### Distribution.

China, Yunnan Province (Baimang Snowberg).

### Delia nigeriposticrus

Taxon classificationAnimaliaDipteraAnthomyiidae

Xue & Du sp. n.

http://zoobank.org/7EC8D21A-C593-4267-9FF6-2EE7691A547C

Figure 2

#### Type material.

***Holotype.*** China, Yunnan Province, Yulong Snowberg, Big ropeway,4571 m, 29 June 2006, Mingfu Wang Co., ♂(IESNU). ***Paratype*.** China, same data as holotype, 2 ♂♂.

#### Diagnosis.

Frontal vitta almost absent at the narrowest part; *pra* longer than posterior notopleural seta; both mid femur and hind femur with complete rows of *av* and *pv*; sternites V processes with expanded tips, without blunt apical setae; postgonite without setae.

#### Description.

***Holotype male*.** Body length 5.0–5.2 mm.

*Head.* Eye bare; frontal vitta black, line form at the narrowest part; frons as wide as anterior ocellus; frontal vitta with 2 pairs of interfrontal setae; without orbital setae; 7 pairs of frontal setae, situated on lower half of frons; fronto-orbital plate and parafacial with fuscous tomentum; parafacial 1.2 times wider than postpedicel; antenna black, postpedicel 1.5–2.0 times longer than broad; arista pubscent, the longest hair shorter than its basal diameter; vibrissal angle situated behind frontal angle in profile; genal height approx. 1/4 eye height; anterior margin of gena with 2 rows of upcurved subvibrissal setulae; postocular setae extending to ventral surface, epicephalon bare; prementum mostly with gray tomentum, 5.0 times longer than broad; palpus short and black, not more than half length of prementum.

*Thorax.* Black in ground color with brown-gray tomentum; scutum with 3 black vittae, extended to scutoscutellar suture; two rows of hair-like *prstacr*, only a single pair of *post acr* developed, *dc* 2+3, *ial* 0+2; one pair of weak outer posthumeral setae; *pra* longer than posterior notopleural seta; scutellum without spots, ventral margins with some pale setae apically; anterior anepisternal setae absent; notopleuron, basisternum of prosternum, anepimeron, meron, and katepimeron bare; both anterior and posterior spiracles small and fuscous; katepisternal seta 1+2.

*Wing.* Base fuscous, basicosta black; costa setulose only basally on ventral surface; costal spine short; radial node bare, calypters brown yellow; lower calypter approx. 1/3 length of upper one; halter yellow.

*Legs.* Entirely black; fore tibia with single medial *p*; mid femur with a complete row of *av*, becoming shorter apically, a complete row of long and dense *pv*, becoming longer mediately, 1.8 times as long as its diameter, 1–2 preapical *pd*;mid tibia with one super-medial *pd* and two *pv*; hind femur with complete rows of *av* and *pv*, becoming longer apically; hind tibia with a row of *av* (approx. 9–10), a row of *ad* (approx. 7–8), a row of *pd* (three strong) and a complete row of *pv*, becoming shorter apically; fore tarsus longer than tibia, all claws and pulvilli longer than tarsomere 5.

**Figure 2.**
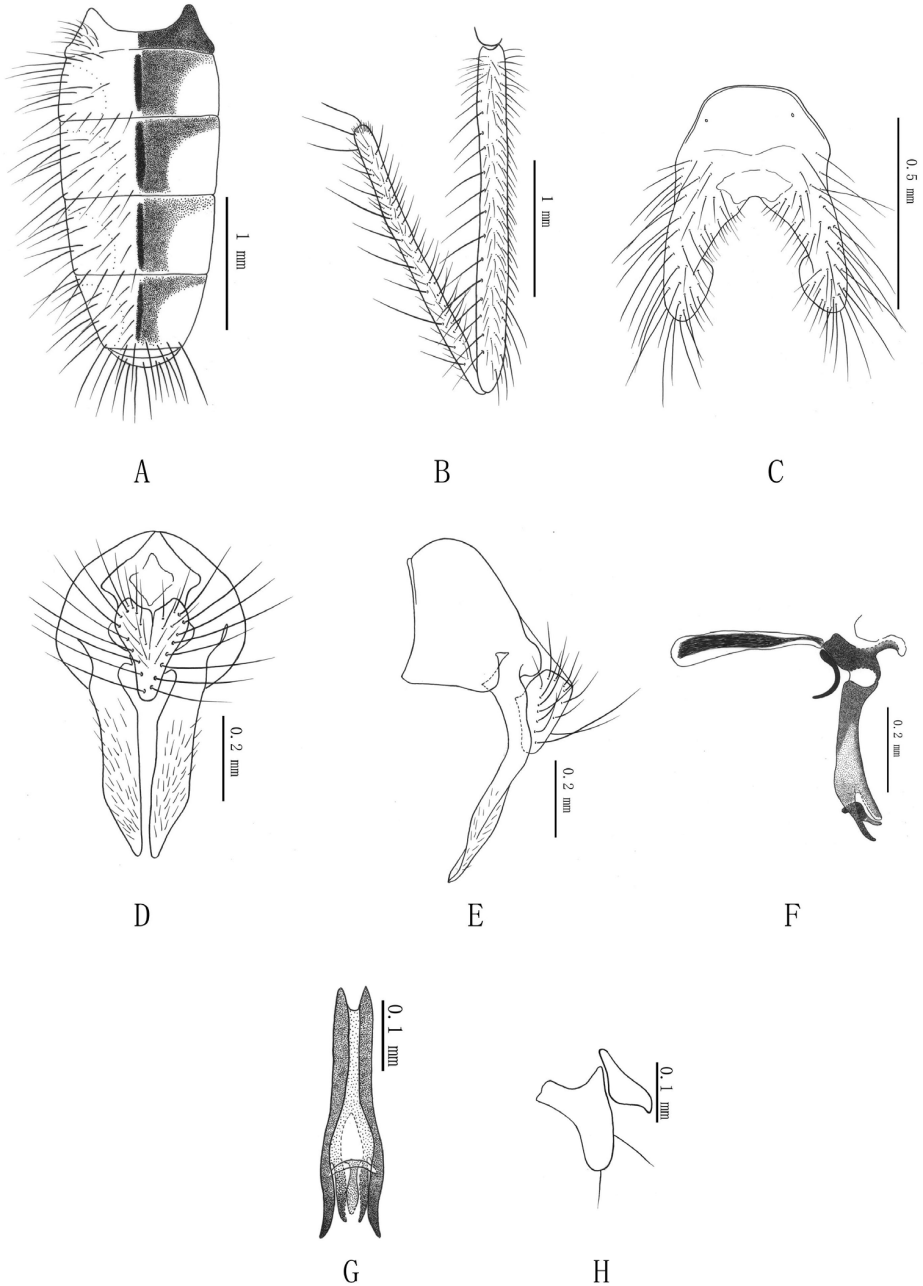
*Delia nigeriposticrus* Xue & Du, sp. n. (male). **A** Abdomen in dorsal view **B** Hind femur and tibia in posterior view **C** Sternite V in ventral view **D** Epandrium, cerci, and surstyli in posterior view **E** Ditto, left lateral view **F** The hypandrial complex in left lateral view **G** Distiphallus in anterior view **H** Gonites.

*Abdomen.* Black, long flat-shapes in dorsal view (Fig. 2A); all tergites with narrow black vittae in center, lateral surface with dark brown allochroic spot, outer lateral surface with gray tomentum; tergite VI bare; sternite I with dense hairs; sternites V processes with expanded tips. Cercal plate (Fig. 2D) 1.2 times longer than wide, heart-shaped, with narrowly rounded apex; surstyli 2.5 times length of cercal plate, in lateral view (Fig. 2E) strongly bent in basal half; pregonite (Fig. 2H) with 2 setae, postgonite without setae. Aedeagus as Fig. 2F–2G; acrophallus longitudinally directed, supported by a sclerotized bridge between the bases of the free paraphallic processes, the sclerotized bridge is prolonged downwards, forming a membranous process.

***Female.*** Unknown.

#### Remarks.

This new species is similar to *D. fulviposticrus* Li & Deng, 1981 as it has very similar genitalia, but differs from it for its male body length 5.0–5.2 mm; frontal vitta black; prementum 5.0 times longer than broad; legs black; mid femur with a complete row of *av*, becoming shorter apically, a complete row of long and dense *pv*, becoming longer medially, 1.8 times as long as its diameter, 1–2 preapical *pd*; hind femur with a complete row of *pv*, becoming longer apically.

#### Etymology.

The specific name is from the Latin word *niger*, black, referring to its legs being entirely black which differs from those of *Delia fulviposticrus* Li & Deng, 1981 which are yellow legs.

#### Distribution.

China, Yunnan Province (Yulong Snowberg).

### Catalogue of the known species in the *Delia interflua* group

***Delia absidata* Xue & Du, 2008**

*Delia absidata* Xue & Du, 2008. Type locality: China: Yunnan, Shangri-la.

**Distribution**. China: Yunnan Province, Shangri-la, Bitahai.

***D. abstracta* (Huckett, 1965)**

Hylemya (Delia) abstracta Huckett, 1965. Type locality: Canada: North-West Territories.

**Distribution**. Canada; Mongolia.

***Delia brevipalpis* Xue & Zhang, 1996**

*Delia brevipalpis* Xue & Zhang, 1996. Type locality: China: Qinghai, Hoh Xil.

**Distribution**. China: Qinghai, Hoh Xil.

**Note**. According to type species, both pregonite and postgonite without setae.

***Delia conjugata* Deng & Li, 1994**

*Delia conjugata* Deng & Li, 1994. Type locality: China: Sichuan, Emeishan.

**Distribution**. China: Sichuan, Emeishan.

**Note**. This species is very similar to *D. stenostyla*, but sternites V processes with narrowly rounded apices, without expanded tips; cercal plate more narrower, with a pair of long distal setae; surstyli in caudal view not expanded medially.

***Delia duplicipectina* Fan in Fan & Zheng, 1993**

*Delia duplicipectina* Fan, 1993. Type locality: China: Sichuan, Xiangcheng.

**Distribution**. China: Sichuan, Xiangcheng.

**Note**. According to type species description, the sternite V processes have broadly rounded apices, without expanded tips.

***Delia fulviposticrus* Li & Deng, 1981**

*Delia fulviposticrus* Li & Deng, 1981. Type locality: China: Sichuan, Emeishan.

**Distribution**. China: Sichuan, Emeishan.

***Delia interflua* (Pandellé, 1900)**

*Chortophila interflua* Pandellé, 1900. Type locality: France: Hautes-Pyrénées, Arrens.

*Chortophila flavisquama* Stein, 1916. Type localities: Germany: Treptow; Austria: Insbruck; Sweden.

*Chortophila setitibia* Stein, 1916. Type localities: Yugoslavia: Istria; Austria: Schneeberg in Krain.

*Hylemyia latifasciata* Ringdahl, 1926. Type locality: Sweden: Jämtland.

*Delia karasawana* Suwa, 1974. Type locality: Japan: Honshû, Nagano-ken, Mt. Hodaka.

**Distribution**. China (Sichuan, Qinghai); Austria; Switzerland; The Czech Republic; Slovakia; Germany; France; Great Britain; Hungary; Italy; Poland; Sweden.

***D. kullensis* (Ringdahl, 1933)**

Hylemya (Delia) kullensis Ringdahl, 1933. Type locality: Sweden: “Kullaberg in Schonen”.

**Distribution**. Sweden; Czech Republic; Slovakia.

***D. kumatai* Suwa, 1977**

*Delia kumatai* Suwa, 1977. Type locality: Nepal: Bangel Kharka.

**Distribution**. Nepal.

***Delia nemostylata* Deng & Li, 1984**

*Delia nemostylata* Deng & Li, 1984. Type locality: China: Sichuan, Emeishan.

**Distribution**. China: Sichuan, Emeishan.

***Delia pansihirta* Jin & Fan in Jin et al. 1981**

*Delia pansihirta* Jin & Fan, 1981. Type locality: China: Gansu, Wenxian.

**Distribution**. China: Gansu, Wenxian.

**Note**. A body covered with more hair is a characteristic that distinguishes this species from other species of this species group.

***Delia spicularis* Fan in Fan et al. 1984**

*Delia spicularis* Fan, 1984. Type locality: China: Qinghai, Yushu.

**Distribution**. China: Qinghai, Yushu.

***Delia stenostyla* Deng & Li, 1994**

*Delia stenostyla* Deng & Li, 1994. Type localities: China: Sichuan, Emeishan; Songpan; Maowen.

**Distribution**. China: Sichuan, Emeishan; Songpan and Maowen.

***Delia subinterflua* Xue & Du, 2008**

*Delia subinterflua* Xue & Du, 2008. Type localities: China: Sichuan, Balangshan; Yunnan, Yulong Snowberg and Baimang Snowberg.

**Distribution**. China: Yunnan Province, Mt. Yulong, Big Ropeway; Sichuan, Mt. Balang.

## Supplementary Material

XML Treatment for Delia subnemostylata

XML Treatment for Delia nigeriposticrus
